# Soil properties drive a negative correlation between species diversity and genetic diversity in a tropical seasonal rainforest

**DOI:** 10.1038/srep20652

**Published:** 2016-02-10

**Authors:** Wumei Xu, Lu Liu, Tianhua He, Min Cao, Liqing Sha, Yuehua Hu, Qiaoming Li, Jie Li

**Affiliations:** 1 Plant Phylogenetics and Conservation Group, Center for Integrative Conservation, Xishuangbanna Tropical Botanical Garden, Chinese Academy of Sciences, Kunming, Yunnan 650223, P. R. China; 2 Department of Environment and Agriculture, Curtin University, PO Box U1987, Perth, WA 6845, Australia; 3 Key Laboratory of Tropical Forest Ecology, Xishuangbanna Tropical Botanical Garden, Chinese Academy of Sciences, Menglun, Yunnan 666303, P. R. China; 4 University of Chinese Academy of Sciences, Beijing 100049, P. R. China

## Abstract

A negative species-genetic diversity correlation (SGDC) could be predicted by the niche variation hypothesis, whereby an increase in species diversity within community reduces the genetic diversity of the co-occurring species because of the reduction in average niche breadth; alternatively, competition could reduce effective population size and therefore genetic diversity of the species within community. We tested these predictions within a 20 ha tropical forest dynamics plot (FDP) in the Xishuangbanna tropical seasonal rainforest. We established 15 plots within the FDP and investigated the soil properties, tree diversity, and genetic diversity of a common tree species *Beilschmiedia roxburghiana* within each plot. We observed a significant negative correlation between tree diversity and the genetic diversity of *B. roxburghiana* within the communities. Using structural equation modeling, we further determined that the inter-plot environmental characteristics (soil pH and phosphorus availability) directly affected tree diversity and that the tree diversity within the community determined the genetic diversity of *B. roxburghiana*. Increased soil pH and phosphorus availability might promote the coexistence of more tree species within community and reduce genetic diversity of *B. roxburghiana* for the reduced average niche breadth; alternatively, competition could reduce effective population size and therefore genetic diversity of *B. roxburghiana* within community.

The relations between species diversity and genetic diversity have long been proposed and observed^1^; however, such correlations have only received increased attention over the last decade^2^,^3^,^4^,^5^,^6^,^7^,^8^,^9^,^10^,^11^,^12^,^13^,^14^,^15^. Species-genetic diversity correlations (SGDCs) could have important implications for the planning of biodiversity conservation because a positive correlation may result in one level of diversity becoming a surrogate for another level^13^,^16^. The majority of the research on SGDCs suggests that species diversity and the intra-population genetic diversity of co-occurring species within a community are positively correlated as result of parallel effects in the environment on both levels of diversity^5^,^7^,^17^. At a regional scale, the neutral ecological processes such as ecological/genetic drift and immigration of species and genes are likely to be the primary drivers of the positive correlation between species diversity and genetic diversity^3^,^5^,^17^,^18^. Moreover, natural or anthropogenic disturbances can have similar effects on species and genetic diversity within a community^4^,^19^,^20^,^21^, although in one recent study, the reaction was dissimilar to disturbance for species and genetic diversity in a riparian forest^11^.

When the local characteristics influence the two levels of diversity in parallel, a positive correlation occurs^5^. Negative SGDCs, however, are reported less frequently in the literature^15^. Vellend *et al*. summarized the causal effects between species and genetic diversity within communities, but more studies are required to investigate the mechanisms of how one level of diversity affects the other level^5^. The classical niche variation hypothesis (NVH) posits that the breadth of the niches of species should be greater in species-poor than species-rich communities; therefore, if greater niche breadth indicates greater genetic diversity, then an increase in species diversity will negatively affect the genetic diversity of co-occurring species within a community or vice versa^5^,^8^,^22^,^23^. Alternatively, adding species to a community could intensify competition and consequently reduce average effective population size of the component species. If the reduction in effective population size is severe, genetic drift alone could lead to a decrease of genetic diversity at both neutral and non-neutral traits^5^.

Over the last decade, the majority of research on SGDCs concentrated on how neutral processes or disturbances affect parallel species diversity and genetic diversity^3^,^17^,^18^,^19^,^20^,^21^, whereas the research on the influence of deterministic processes on biodiversity potentially have provided significant insight into the origin and the maintenance of different levels of biodiversity^7^. The relationships between the distributions of plants and soil properties have long attracted the interest of ecologists^24^,^25^,^26^,^27^,^28^,^29^; however, relatively little is known about the effect of soil properties on the genetic diversity of a population of a focal species. Research that addresses this gap in our understanding could provide new perspectives on the consequences of global ecological issues such as atmospheric nitrogen deposition and eutrophication on the biodiversity of diverse ecosystems.

In this study, we established 15 plots (40 m × 40 m) within a 20 ha forest dynamics plot (FDP) in the Xishuangbanna tropical seasonal rainforest (Fig. S1). We surveyed the tree diversity of the community, evaluated the genetic diversity of a common tree species *Beilschmiedia roxburghiana* (Lauraceae) using microsatellite DNA markers, and measured the environmental properties (soil nutrients and topography) of each plot. Our goal was to answer two fundamental questions: 1) Are the tree diversity of a community and the genetic diversity of a common tree species correlated? 2) What are the possible drivers of the SGDC pattern in these species-diverse communities?

## Results

Across the 15 plots, the soil properties were highly variable (Table S1). The content of extractable phosphorus (EP) varied sixfold and ranged from 1.78 to 10.06 mg/kg. A total of 311 tree species with DBH (diameter at 1.3 m height) >1 cm were recorded within the 15 plots of 1600 m^2^ each (Appendix S1). The total tree abundance among the plots varied from 680 to 1186 with an average of 902 trees per plot. An average of 116 tree species were recorded in each plot and the rarefied tree species richness ranging from 90 to 124. The rarefied allelic richness (R_a_) varied from 1.90 to 4.48 across the 15 plots (Table 1). The genetic differentiation of *B. roxburghiana* was significant among the plots (F_ST_ = 0.097, *P* = 0.001). The soil properties within each plot were not correlated with the effective population size of *B. roxburghiana* (Table 2, Fig.S2a, b); while the rarefied tree richness and effective population size of *B. roxburghiana* was negative (*P* = 0.063) within each plot (Fig. S2c).

**Figure 1:**
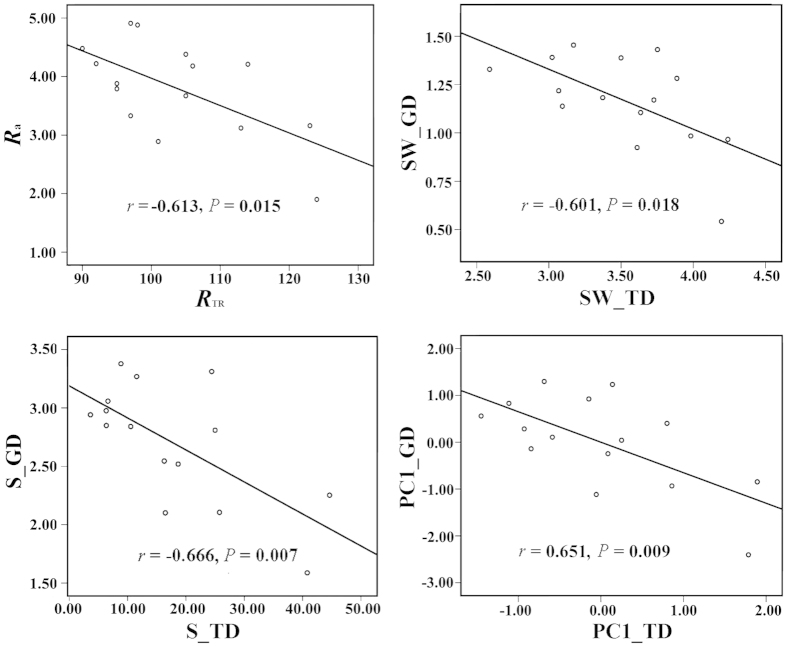
Negative correlation between the genetic diversity of *B. roxburghiana* and the tree species diversity within each plot. *R*_a_, rarefied number of alleles per locus (rarefacted to the smallest sample size of seven); *R*_TR_, rarefied tree richness (rarefacted to the smallest sample size of 680); S_GD & S_TD and SW_GD & SW_TD, inverse Simpson index and Shannon-Wiener index, respectively, for genetic diversity of *B.roxburghiana* and tree diversity within the plots, respectively. PC1_TD represents the first component (93.87% of the variance explained) from the PCA analysis that was based on the correlation matrix of *R*_TR_, SW_TD and S_TD; and PC1_GD represents the first component (96.91% of the variance explained) from the PCA analysis that was based on the correlation matrix of *R*_a_, SW_GD and S_GD. Both PC1_TD and PC1_GD were positively correlated with the primary variables, with *P* < 0.001. The PC1_TD and PC1_GD were used as comprehensive measures to represent the tree diversity and the genetic diversity of *B. roxburghiana* within each plot, respectively.

**Figure 2:**
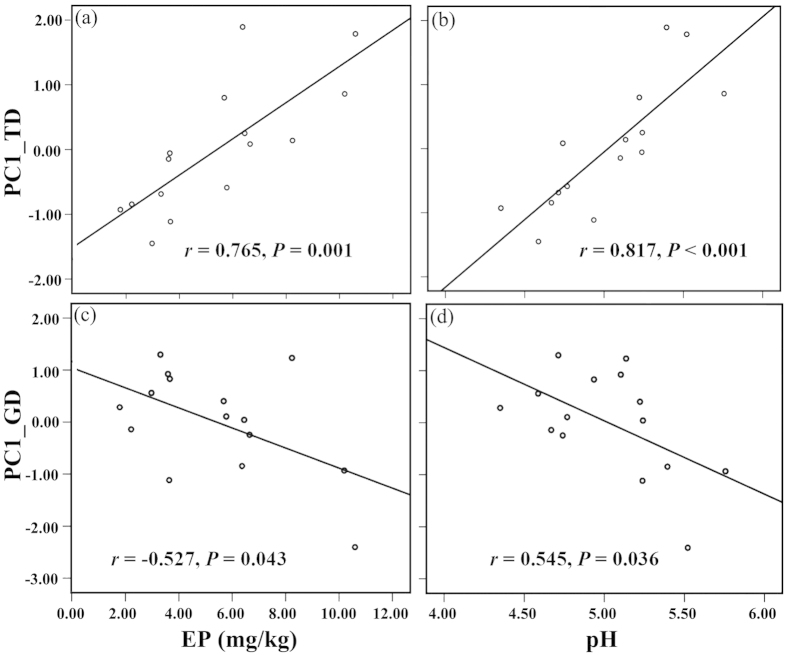
Contrasting patterns of the soil pH and EP on the tree diversity and the genetic diversity of *B.roxburghiana* within each plot.

From the Pearson correlation analysis, a significant negative correlation was found between the specific pair of indexes at the species and the genetic levels (Fig. 1). From further analysis, the availability of soil phosphorus and the pH had contrasting effects on the tree diversity and genetic diversity of *B. roxburghiana* within the community (Table 2, Fig. 2). Plots with increased soil phosphorus availability, had significantly increased tree species diversity (Fig. 2a), whereas the genetic diversity of *B. roxburghiana* decreased (Fig. 2c). A similar effect of soil pH on species and genetic diversity was also observed (Fig. 2b,d). The topography of the plot primarily influenced the soil pH (r =-0.735, *P* < 0.01), whereas the soil pH was correlated with phosphorus availability (r = 0.778, *P* < 0.01) (Table S2, Fig. S3). None of the biodiversity measures were significantly correlated with the topographical variables (Table S3).

**Figure 3:**
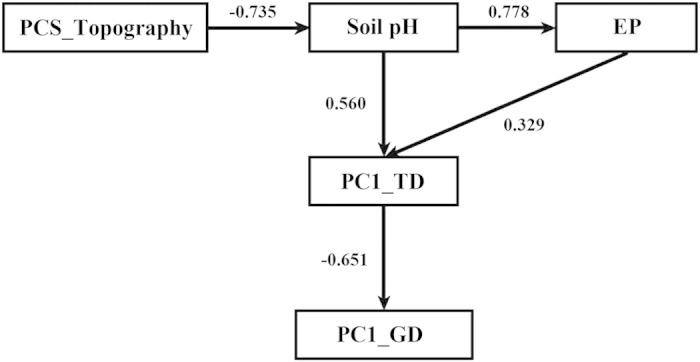
Optimized structural model showing the effect network among the topography, soil properties, tree diversity and genetic diversity of *B. roxburghiana* within each plot. The numbers next to the arrows are the standardized direct effects. All connection pathways are significant at *P* < 0.05. N = 15, *df* = 5, χ^2^ = 1.915, and *P* = 0.861; CFI (comparative fit index) = 1; GFI (goodness of fit index) = 0.951; and RMSEA (root mean square error of approximation) < 0.01. The PCS_ Topography was calculated as the measure of topography using only the two components with eigenvalues above 1 (79.38% of the variance explained; the first principle component primarily explained elevation, slope and aspect, and the second principle component explained convex; also see Methods) in the PCA analysis. PC1_TD represents the tree diversity and PC1_GD represents the genetic diversity of *B. roxburghiana* within each plot.

A hierarchical effect among the environmental properties, tree diversity and genetic diversity of *B. roxburghiana* within the plots was identified with the structural equation modeling (SEM) analysis. A number of SEM models were tested in our study, and the best-fit model (RMSEA <0.01; CFI = 1.0; and GFI = 0.951) was selected (Figs 3 and S4). The best-fit model suggested that the increase in tree diversity within a plot was directly affected by the elevated soil pH and available phosphorus, whereas the increase in species diversity directly depleted the genetic diversity of *B. roxburghiana* (Fig. 3). The SEM analysis also revealed that the variation in soil pH was a direct consequence of the topographic variation and that soil pH determined the availability of phosphorus (Fig. 3).

## Discussion

A positive SGDC was the typical result found in previous studies that examined the parallel effects of the environment on the two levels of diversity^4^,^5^,^7^,^15^,^19^; however, negative SGDCs are observed less often, and the drivers of negative SGDCs are rarely discussed in the literature on empirical studies^13^. In our study, a negative correlation was found between the tree diversity and the genetic diversity of *B. roxburghiana* (Fig. 1). Based on SEM analysis, the environmental characteristics (soil pH and phosphorus availability) effected tree diversity within the community and ultimately led to the negative SGDC that was observed in our study (Fig. 3). A negative SGDC driven by environmental characteristics was also previously reported^8^.

The “humped-back curve” predicts the relationship between resource availability and species diversity^30^,^31^; species richness is low at low nutrient levels, increases to a peak at intermediate levels and then declines gradually at high nutrient levels. We observed significant positive relationships among phosphorus availability, soil pH and tree diversity within our plots. The phosphorus deficiency and the strong acidity of the soil were likely important factors that limited the survival of many tree species in the Xishuangbanna tropical seasonal rainforest. Xue *et al*. found that phosphorus is the most limiting nutrient in the Xishuangbanna tropical seasonal rainforest and that the acidic soils further limit phosphorus availability^32^. Based on the SEM analysis, the soil pH was primarily determined by the topography within our plots, and the plots that were in the valley (lower elevation and convex) generally had relatively high soil pH values (Table S2, Figs 3 and S3). The topography can influence the hydrology and the soil pH within a community^33^,^34^. An increase in the pH of the acidic soils could release more of the phosphorus that was fixed by iron or aluminum ions when the soil pH was relatively low^32^,^35^. Moreover, soil pH also directly effects plant growth^35^.

From the pairwise correlation analyses, negative correlations were found between the soil factors (soil pH and phosphorus availability) and the genetic diversity of *B. roxburghiana* (Table 2). An increase in the soil pH and phosphorus availability could have direct and negative effects on the individual *B. roxburghiana*, with fewer individuals surviving in an environment with relatively high phosphorus availability and soil pH values. Such a reduction in the effective population size could therefore decrease the genetic diversity. However, this explanation was less likely in our study because the effective population size of *B. roxburghiana* was not correlated with the soil pH (r = −0.263, *P* = 0.344) or EP (r = −0.214, *P* = 0.444) (Fig. S2a, b), whereas the soil factors and tree richness were positively correlated (Table 2). Thus, to explain the negative correlations between the two soil factors and the genetic diversity of *B. roxburghiana*, other mechanisms must be examined.

The best-fit SEM model further indicated that increases in the soil pH and phosphorus availability promoted tree diversity, and the increase in tree diversity within the community resulted in a decrease in the genetic diversity of *B. roxburghiana* (Fig. 3). An increase in tree diversity within a community could decrease the effective population sizes of the component species, because the carrying capacity of the system might be limited. Consequently, a small-sized population of *B. roxburghiana* could contain less genetic diversity^5^. We revealed a negative correlation (*P* = 0.063) between the tree richness in the plot and the effective population size of *B. roxburghiana* within community, and also a significant positive correlation between effective population size of *B. roxburghiana* and its genetic diversity (Fig. S2c, d); With increased tree species diversity, competition could reduce the effective population size and therefore genetic diversity of *B. roxburghiana* within community^5^.

The third explanation for the negative SGDC is found in the niche variation hypothesis^5^,^8^,^22^. With increases in the soil pH and phosphorus availability, more tree species coexisted within the community, and with more tree species, the genetic diversity of *B. roxburghiana* decreased because the average niche breadth was reduced. Recently, Yang *et al*.^36^ reported that a the deterministic processes could be the primary driver in the assembly of communities within the FDP. The connections between the environment and the trees, as well as species interactions, likely played large roles in the assembly of the forest community within the FDP.

Using the niche variation hypothesis to explain a negative SGDC typically assumes that high genetic diversity is an indication of large niche breadth^5^,^22^. The SSR markers are generally assumed to be neutral, and it is notable that the individual SSR alleles may not be related to niche breadth. The genetic diversity of a focal species, particularly the dominant species as measured by neutral molecular markers, might have important ecological consequences^37^,^38^,^39^. In a recent study, the different SSR genotypes were also related to morphological and physiological variation in *Zostera marina*^37^. It is likely that neutral genetic diversity may substitute for the level of adaptive genetic variation within a population, and different genotypes may prefer to survive within a specific environment. However, the recent meta-analysis revealed that there was no overall association between neutral genetic diversity and measures of ecological structure^40^; thus, invoking niche variation hypothesis as an alternative mechanism to explain the current negative SGDC, further studies are needed to link the neutral genetic diversity and niche breadth of *B. roxburghiana* along an environmental gradient within the Xishuangbanna tropical seasonal rainforest.

In the previous studies on SGDCs, the general assumption is that the forces that maintain species diversity and genetic diversity are similar, as first proposed by Antonovics^1^, and positive patterns are reported that are consistent with this assumption. The neutral processes such as ecological/genetic drift and immigration of species and genes likely drive the positive SGDCs within the discrete sampling units such as islands, forest fragments and ponds^3^,^14^,^18^,^20^. However, environmental characteristics can also cause a positive SGDC^7^,^9^,^41^. For example, Marshall & Camp concluded that environmental characteristics were positively correlated with both the richness of lungless salamander species (Plethodontidae) and the respective allelic richness^41^. However, based on our results, environmental characteristics can also generate a negative SGDC (Table 2, Figs 1 and 2). Taberlet *et al*. argued that environmental characteristics in glacial refugia likely contribute to the negative and zero SGDCs in the flora of European alpine regions^16^. The topographic variation in the glacial refugia might have promoted species diversity by increasing the coexistence of species, but the topographic variation might also have caused limited gene flow and led to population genetic drift^42^. The results of our study are consistent with the hypothesis that environmental characteristics are important drivers of SGDCs, and whether the pattern is positive or negative depends on the ecosystem context and the community composition^13^.

The connections between species and genetic diversity were first discussed four decades ago^1^, but such connections have only received renewed interest within the last ten years or so, partially because the correlation between the two levels of diversity has important implications for the conservation of biodiversity^13^. A positive SGDC is considered the typical relationship and is based on the tenet of Antonovics that the forces that maintain species diversity and genetic diversity are similar^1^. However, based on recent research and the results of our current study, negative SGDCs also occur^6^,^8^,^23^. As Taberlet *et al*. noted, any type of correlation may be found when testing the genetic diversity of a single species within a community in a SGDC analysis^16^. Consequently, we cannot take it for granted to use species diversity as a surrogate for genetic diversity, or vice versa, in conservation planning^13^.

## Methods

### Study site and focal species

Fifteen plots, each 1600 m^2^ (40 × 40 m), were established within the 20 ha (400 m × 500 m) FDP in the Xishuangbanna tropical seasonal rainforest in south-western China (centred at 21°37′08″ N, 101°35′07″ E)^43^. The FDP is situated within the Indo-Burma biodiversity hot spot^44^ (Fig. S1). The average annual rainfall of the region is 1493 mm. The FDP has a laterite soil that developed from siliceous rocks^45^. The elevation of the 15 plots ranges from 725 to 837 m. The location of each plot was chosen to include the maximum number of *B. roxburghiana* within the plot.

*Beilschmiedia roxburghiana* Nees is an evergreen, small- to medium-sized tree that grows in the tropical, evergreen, broad leaf forests in south-eastern Xizang Province and Yunnan Province, China, and in north-eastern Myanmar and India. This tree is typically a forest-dwelling species that generally occupies the second and third layers of the canopy and that can reach 20 m in height. Insects pollinate the hermaphroditic flowers, and gravity and vertebrates, such as birds and small mammals, disperse the seeds^46^. The populations of *B. roxburghiana* have become increasingly fragmented in recent years because of the deforestation that is a consequence of the development of rubber tree plantations. In addition to *B. roxburghiana*, the common tree species in the plots include *Pittosporopsis kerrii* (Icacinaceae), *Parashorea chinensis* (Dipterocarpaceae), *Knema furfuracea* (Myristicaceae) and *Garcinia cowa* (Clusiaceae).

### Topography, soil nutrient analyses and species diversity survey

For each of the 15 plots, the elevation, aspect, slope and convex were calculated using the procedures described in Liu *et al*.^47^. The soil nutrients and properties that were measured were ammonium nitrogen (AN), extractable phosphorus (EP), exchangeable potassium (EK), pH, organic matter (OM), total nitrogen (TN), total phosphorus (TP), total potassium (TK) and soil bulk density (BD). The soil nutrient analyses followed the protocols in Hu *et al*.^48^. In each plot, all trees with a DBH >1 cm were surveyed and recorded. Three species diversity indices (Tree richness = TR, number of tree species within community; the Shannon-Wiener index = SW_TD, calculated as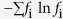, where *f*_i_ is the number of the focal tree species divided by the number of samples within the community; and the inverse Simpson index = S_TD, calculated as 1/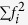) were calculated^49^. The rarefied tree species richness (*R*_TR_) was calculated using a rarefaction procedure implemented with R statistical software package (R Development Core Team 2013).The Xishuangbanna Station for Tropical Rain Forest Ecosystem Studies of the Chinese Academy of Sciences provided all primary data for this section of the FDP.

### Genetic diversity of *B. roxburghiana*

The genetic diversity of *B. roxburghiana* in each of the 15 plots was measured using ten pairs of microsatellite DNA primers. We considered each plot a “population”, although the distribution of *B. roxburghiana* is more or less continuous in the FDP. An average of 11 samples (7–22) were genotyped. The protocols for the microsatellite genotyping and the primer sequences were described in Liu *et al*.^50^. Briefly, the total genomic DNA was extracted using a modified cetyltrimethylammonium bromide (CTAB) method^51^. Following PCRs, the PCR products were separated in an ABI 3730 sequencer (Applied Biosystems, Carlsbad, CA, USA), and the fragment lengths were analysed using ABI GeneMapper software version 3.7 (Applied Biosystems). To measure the genetic diversity in each plot, the allelic richness (N_a_), the Shannon-Wiener index (SW_GD, calculated as 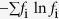, where *f*_i_ is the frequency of the ith allele for the population), and the inverse Simpson index (S_GD, 1/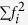) were calculated using GenAlEx6.5^52^. To eliminate the effect of uneven sample size on the measurement of N_a_, we calculated the rarefied allelic richness (*R*_a_) using a rarefaction procedure implemented with HP-rare1.0^53^, and this procedure resampled individuals from populations with sample sizes larger than the minimum to calculate the allelic richness expected when the smallest samples were taken from each population. We estimated effective population size (*N*_e_) of *B. roxburghiana* within each plot using an updated version of the heterozygote-excess method^54^, as implemented in NeEstimator V2^55^; We assumed a random mating model and calculated the estimates using the threshold allele frequencies of *P*_crit_ = 0.05 for excluding rare alleles. To clarify the degree of genetic differentiation among the populations, the F_ST_ was calculated, and the significance was obtained with 999 permutations using GenAlEx6.5^52^.

### Statistical analyses

The Shapiro-Wilk test was first implemented to evaluate the normal distribution of all variables; TK and *N*_e_ were consequently log-transformed to improve normality. In our study, four data sets were developed (topography, soil nutrients, tree diversity and genetic diversity of *B. roxburghiana* within the plots), and each data set contained several variables that were possibly correlated with one another. Therefore, we analysed these data sets with principal component analysis (PCA) based on their correlation matrices. The principal components score (PCS) was calculated for the topographical variables and the soil nutrients because two or three principal components had eigenvalues above 1 during the PCA analysis. The PCS was calculated as follows: PCS = (λ_1_/(λ_1_ + λ_2_+λ_3_)) × F_1_ + (λ_2_/(λ_1_ + λ_2_ + λ_3_)) × F_2_ + (λ_3_/(λ_1_ + λ_2_ + λ_3_)) × F_3_, where λ_1_, λ_2_, and λ_3_ are the eigenvalues of the three components and F_1_, F_2_, and F_3_ are the first three components; λ_3_ and F_3_ equal zero when only two components have eigenvalues above 1 during a PCA analysis. We first explored the pairwise correlations between topography, soil nutrients, tree diversity and genetic diversity of *B. roxburghiana* in the plots using simple Pearson correlation analysis, and the significance differences were corrected for multiple comparisons following the Bonferroni procedure. An analysis with SEM was used to further generate and explore the model and to infer the causal correlations among topography, soil nutrients, tree diversity and genetic diversity of *B. roxburghiana*. To begin, we constructed three conceptual SEM models of the expected multivariate relationships based on theoretically developed hypotheses of the interactions among the variables (Fig. S4)^5^.The best-fit model in this study included all significant connecting pathways with CFI (comparative fit index) >0.9; GFI (goodness of fit index) >0.9 and RMSEA (root mean square error of approximation) <0.02. The Shapiro-Wilk test, PCA and Pearson correlation analyses were implemented using the SPSS statistical software package 16.0, and the SEM analysis was conducted using Amos 20.0 (SPSS Inc., Chicago, IL, USA). The Bonferroni corrections were conducted using the R statistical software package (R Development Core Team 2013). The significance level was *P* < 0.05.

## Additional Information

**How to cite this article**: Xu, W. *et al*. Soil properties drive a negative correlation between species diversity and genetic diversity in a tropical seasonal rainforest. *Sci. Rep.* **6**, 20652; doi: 10.1038/srep20652 (2016).

## Supplementary Material

Supplementary Information

## Figures and Tables

**Table 1:** Summary of the genetic diversity of the 15 populations of *B. roxburghiana* and the tree diversity with DBH >1 cm within each plot.

Code	PS (*N*_e_)	Genetic diversity			Tree diversity		
		*R*_a_	S_GD	SW_GD	TR (*R*_TR_)	S_TD	SW_TD
P1	9 (1.1)	1.900	1.587	0.542	130 (124)	40.792	4.195
P2	7 (2.3)	3.156	2.252	0.966	123 (123)	44.625	4.239
P3	22 (2.8)	3.118	2.105	0.984	123(113)	25.748	3.983
P4	11 (6.8)	4.206	2.809	1.282	123 (114)	25.001	3.886
P5	18 (2.3)	2.892	2.101	0.924	107 (101)	16.469	3.611
P6	7 (3.7)	4.182	2.519	1.170	116 (106)	18.668	3.727
P7	8 (3.4)	3.675	2.545	1.105	117 (105)	16.284	3.635
P8	8 (13.1)	4.914	3.311	1.431	109 (97)	24.413	3.752
P9	9 (7.4)	4.882	3.378	1.454	110 (98)	8.863	3.170
P10	13 (4.8)	4.376	3.268	1.388	120 (105)	11.557	3.499
P11	13 (2.6)	3.332	2.849	1.138	111 (97)	6.358	3.093
P12	7 (3.5)	3.790	2.841	1.182	116 (95)	10.546	3.373
P13	7 (3.3)	3.878	2.976	1.218	111 (95)	6.333	3.068
P14	15 (3.9)	4.221	2.941	1.329	115 (92)	3.630	2.589
P15	14 (5.4)	4.480	3.058	1.390	105 (90)	6.636	3.022

PS, census population size of *B. roxburghiana* in each plot; *N*_e_, effective population size; *R*_a_, number of alleles per locus (rarefacted to the smallest sample size of seven); TR, tree richness; *R*_TR_, rarefied tree richness (rarefacted to the smallest sample size of 680); S_GD & S_TD and SW_GD & SW_TD, inverse Simpson index and Shannon-Wiener index, respectively, for genetic diversity of *B. roxburghiana* and tree diversity within the plots, respectively. Additionally, see Methods for details of the calculations for the biodiversity measures.

**Table 2:**
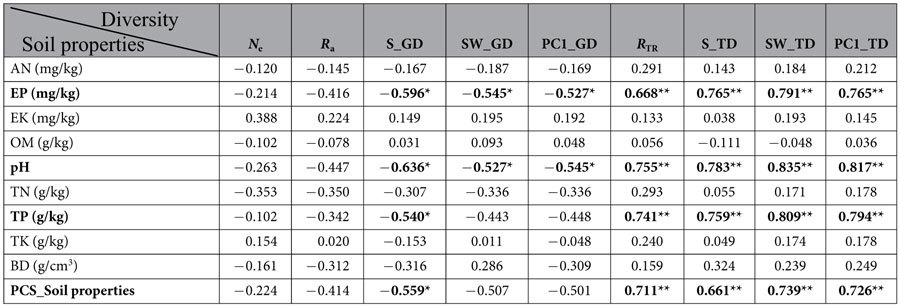
Pairwise coefficients of correlation showing the effects of soil properties on the tree diversity and the genetic diversity of *B. roxburghiana* within each plot.

AN, ammonium nitrogen; EP, extractable phosphorus; EK, exchangeable potassium; OM, organic matter; TN, total nitrogen; TP, total phosphorus; TK, total potassium; and BD, soil bulk density. *N*_e_ and TK were log-transformed to improve normality. PCS_Soil properties were calculated as the measure of soil nutrient availability within each plot using only the first three components in the PCA analysis with eigenvalues above 1 (88.50% of the variance explained, also see Methods). All significance was determined for the Bonferroni corrections.

*Correlationis significant at 0.05, **Correlation is significant at 0.01 (2-tailed).
