# Supplementary material for: Soil properties drive a negative correlation between species diversity and genetic diversity in a tropical seasonal rainforest
Source: Sci Rep. 2016 Feb 10;6:20652. doi: 10.1038/srep20652 (PMC4748317; doi:10.1038/srep20652)
Supplement: Supplementary Information [file srep20652-s1.pdf]

Soil properties drive a negative correlation between species diversity and genetic diversity in a tropical seasonal rainforest

Wumei Xu, Lu Liu, Tianhua He, Min Cao, Liqing Sha, Yuehua Hu, Qiaoming Li, Jie Li

Supplementary information:

Table S1 Soil nutrients and pH of the 15 plots within the FDP.

Code	AN (mg/kg)	EP (mg/kg)	EK (mg/kg)	OM (g/kg)	pH	TN(g/kg)	TP (g/kg)	TK (g/kg)	BD (g/cm^3)
P1	202.871	10.597	177.840	20.497	5.521	2.031	0.456	9.452	1.162
P2	165.731	6.336	140.289	15.917	5.394	1.702	0.394	8.998	1.181
P3	173.523	10.194	204.579	16.579	5.756	1.803	0.420	11.364	1.238
P4	201.980	5.678	304.415	19.001	5.222	2.038	0.504	18.713	1.123
P5	161.111	3.638	158.177	17.191	5.238	1.826	0.363	11.101	1.168
P6	173.745	6.446	169.916	16.890	5.241	1.760	0.340	11.731	1.155
P7	194.782	6.647	254.104	19.287	4.740	2.013	0.389	12.442	1.067
P8	162.390	8.236	203.091	16.734	5.136	1.554	0.372	8.479	1.222
P9	181.558	3.309	230.422	19.203	4.713	1.806	0.303	10.414	1.082
P10	178.943	3.591	179.225	19.337	5.103	1.910	0.309	8.626	1.106
P11	169.403	2.211	198.868	17.147	4.668	1.778	0.247	9.506	1.213
P12	195.227	5.775	213.366	19.774	4.768	2.033	0.345	10.684	1.126
P13	164.637	1.783	112.716	17.989	4.350	1.742	0.215	9.201	1.130
P14	174.831	2.973	159.261	17.707	4.589	1.771	0.274	10.576	1.167
P15	182.820	3.661	170.558	17.619	4.935	1.798	0.319	9.274	1.127

AN, ammonium nitrogen; EP, extractable phosphorus; EK, exchangeable potassium; OM, organic matter; TN, total nitrogen; TP, total phosphorus; TK, total potassium; and BD, soil bulk density.

Table S2 Pairwise coefficients of correlation between topography and soil properties of each plot.

Topography / Soil properties	Elevation (m)	Slope	Convex	Aspect	PCS_Topography
AN (mg/kg)	-0.060	0.065	0.001	-0.372	-0.172
EP (mg/kg)	-0.231	-0.128	-0.431	-0.370	-0.470
EK (mg/kg)	-0.259	0.045	0.127	-0.447	-0.200
OM (g/kg)	0.059	0.106	-0.033	-0.361	-0.145
**pH**	**-0.520***	-0.315	**-0.689****	-0.423	**-0.735****
TN (g/kg)	-0.031	-0.121	-0.076	-0.363	-0.215
TP (g/kg)	-0.439	-0.278	-0.317	-0.376	-0.489
TK (g/kg)	-0.283	-0.231	0.142	-0.252	-0.138
BD (g/cm^3)	-0.152	0.313	-0.483	0.289	-0.062
PCS_Soil properties	-0.341	-0.173	-0.408	-0.513	-0.457

The PCS_ Topography was calculated as the measure of topography using only the two components with eigenvalues above 1 (79.38% of the variance explained, PC1 primarily explained elevation, slope and aspect, and PC2 explained convex; PC1 = 0.423×elevation′ + 0.210×convex′ + 0.355×slope′ + 0.357×aspect′; and PC2 = -0.144×elevation′ + 0.761×convex′ －0.520×slope′ + 0.241×aspect′, “′” indicate that data were Z-score transformed with a mean of 0 and a standard deviation of 1) in the PCA analysis. All primary variables were positively correlated with PCS_ Topography at $P < 0.01$ except slope (r = 0.408; $P = 0.131$). Note the aspect was transformed and quantized by shade slope (0.3, approximately 0-45°, approximately 315-360°), semi-shade slope (0.5, approx. 45-90°, approx. 270-315°), semi-sunny slope (0.8, approx. 90-135°, approx. 225-270°) and sunny slope (1.0, approx. 135-225°) to show the light and temperature gradient[1].

* Correlation is significant at $P < 0.05$ (2-tailed).

** Correlation is significant at $P < 0.01$ (2-tailed).

Table S3 Pairwise coefficients of correlation showing the effects of topography on tree diversity and genetic diversity of *B. roxburghiana* within each plot.

Diversity / Topography	R_a	S_GD	SW_GD	PC1_GD	R_{TR}	S_TD	SW_TD	PC1_TD
Elevation (m)	-0.156	-0.045	0.012	-0.030	-0.296	-0.344	-0.252	-0.257
Slope	0.158	0.240	0.368	0.266	-0.314	-0.343	-0.220	-0.240
Convex	0.424	0.409	0.414	0.422	-0.427	-0.364	-0.354	-0.427
Aspect	-0.013	0.052	0.080	0.040	-0.443	-0.422	-0.207	-0.384
PCS_Topography	0.185	0.245	0.321	0.254	-0.486	-0.508	-0.373	-0.491

*. Correlation is significant at $P < 0.05$ (2-tailed).

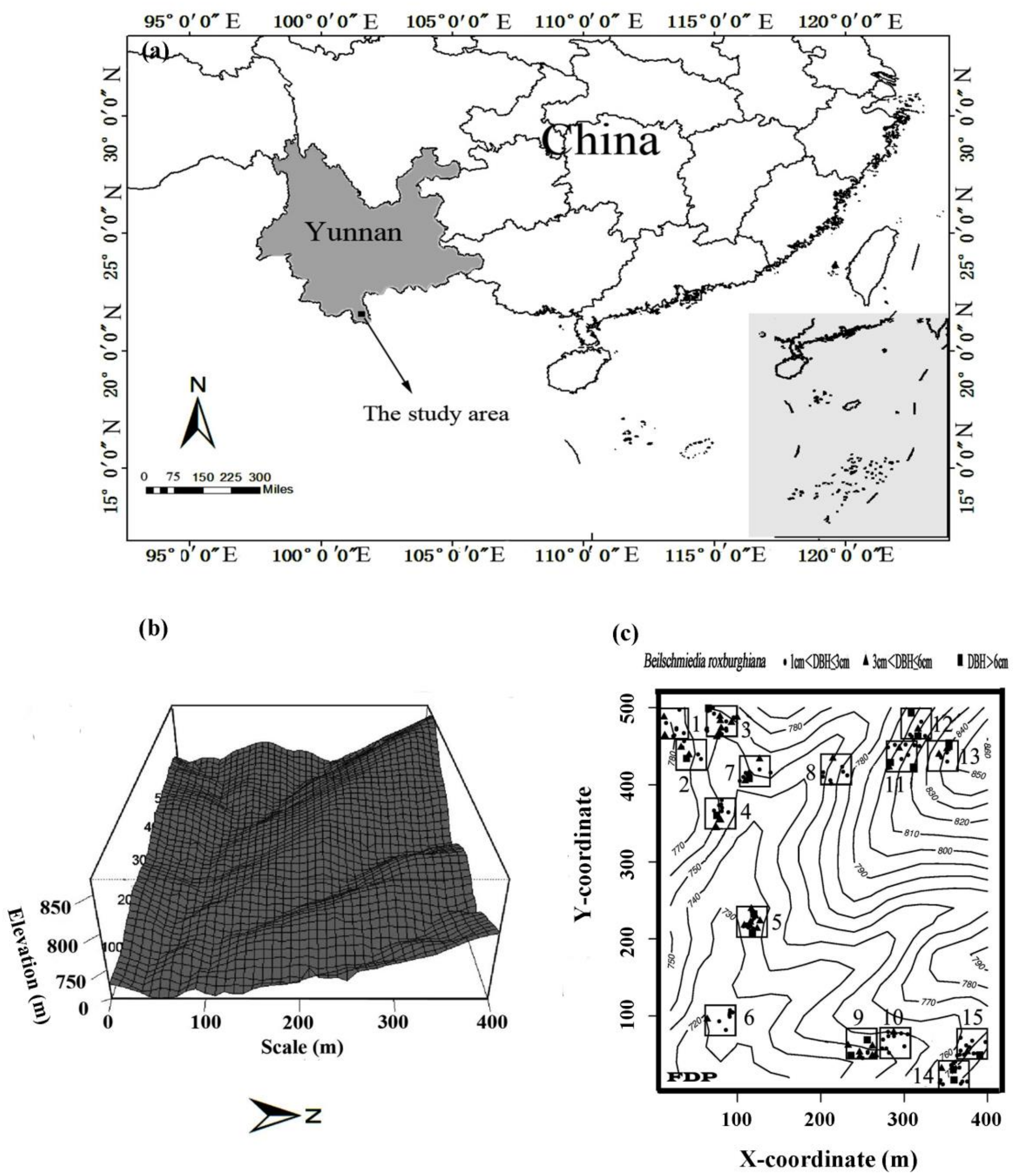

Figure S1 The study area (a), topographic map of the 20 ha FDP (b), and the plots within the FDP in Xishuangbanna (c). Note that the geographic map of the study area was created with Arcgis 10.0 (http://www.esri.com/apps/products/arcgis/eval10/evalhelp/index.cfm). The three-dimensional map of the 20 ha FDP and the plot map within the FDP were created using the R statistical software package (http://www.r-project.org/). The full figure was generated with Adobe Photoshop CS 6 (http://www.adobe.com/content/dotcom/cn/products/photoshop.html).

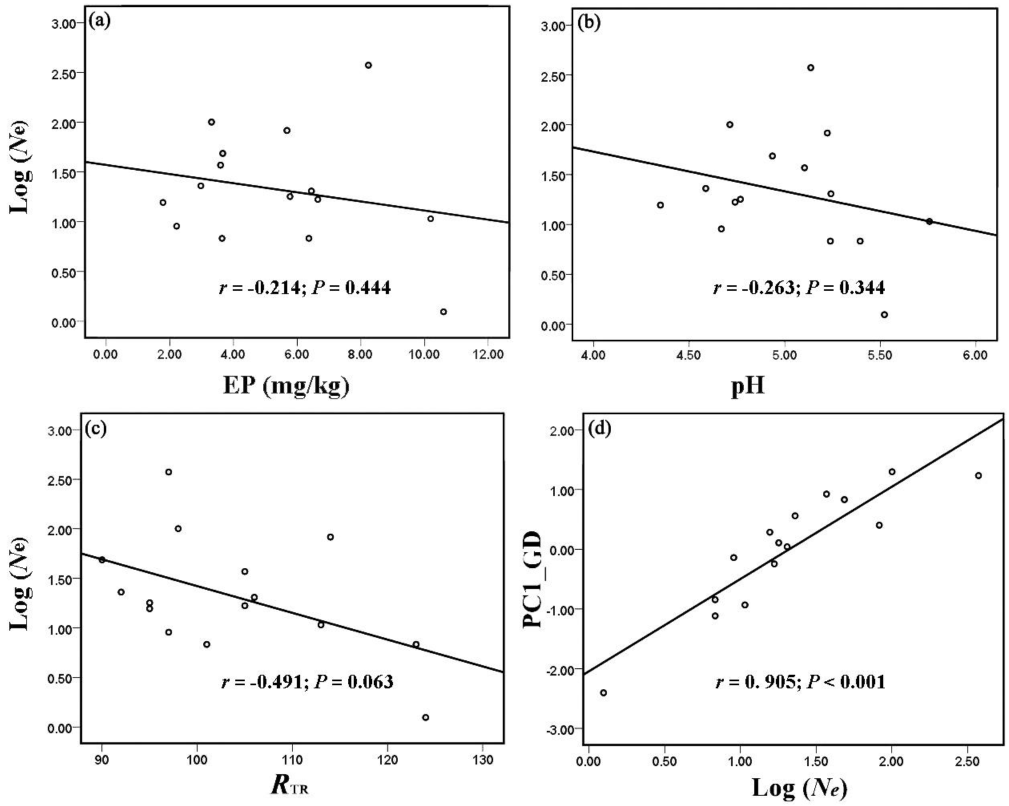

Figure S2 Connections between soil properties (soil pH and EP) and the effective population size of *B. roxburghiana* (N_e, Log-transformed to improve normality); and the connections between the rarefied tree richness and N_e and its genetic diversity (PC1_GD) within each plot.

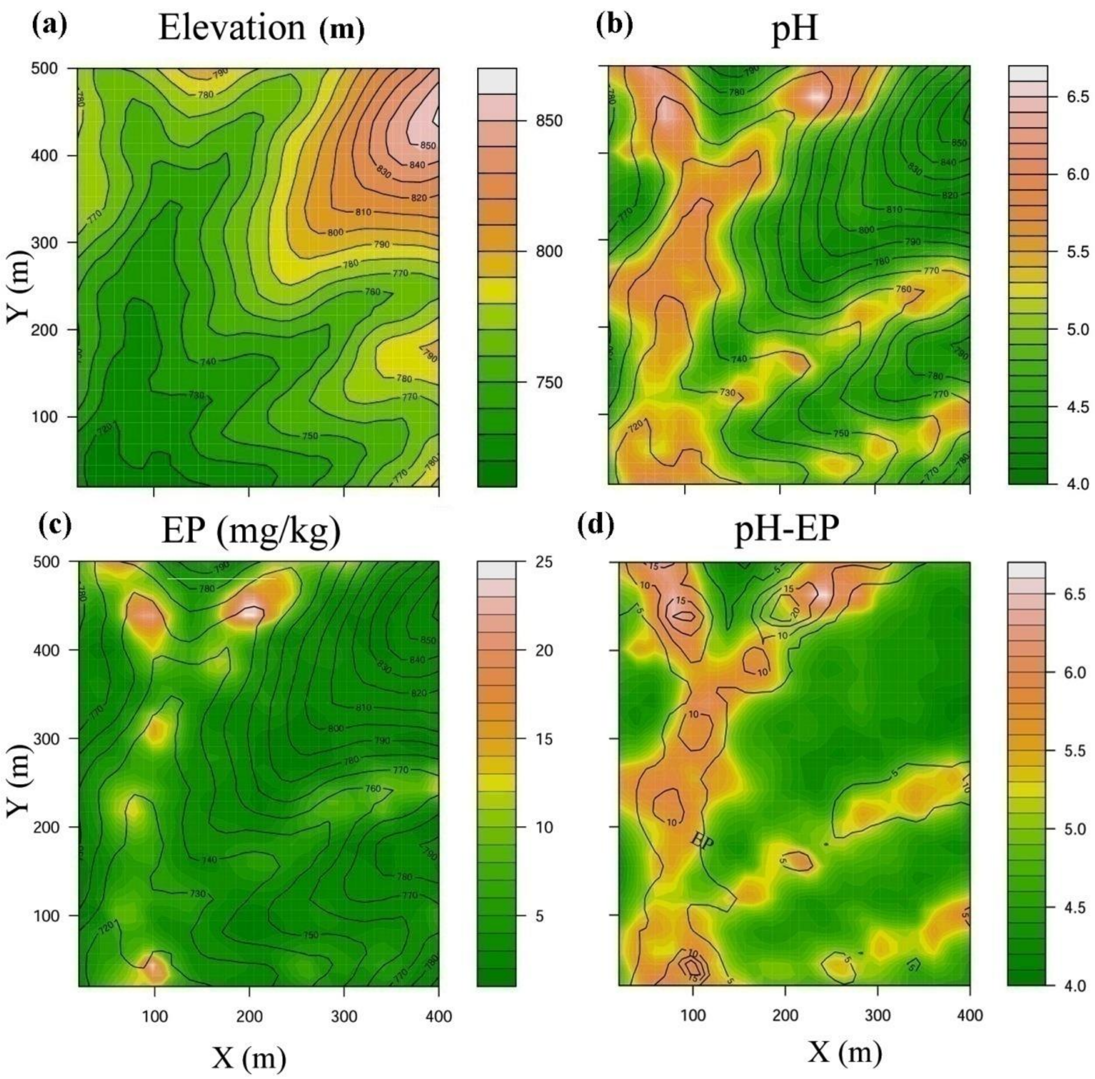

Figure S3 Topographical map of the 20 ha FDP and the soil pH and the distribution of phosphorus availability across the FDP. These figures indicate the sites near the stream (in the valley) in which the soils shared a high pH (b) and the sites with high soil pH that typically shared high EP (d) across the 20 ha FDP. Note that figure S3a, b, c & d were created using the R statistical software package (Version: Ri386 3.2.0, http://www.r-project.org/). The full figure was generated with Adobe Photoshop CS 6 (http://www.adobe.com/content/dotcom/cn/products/photoshop.html).

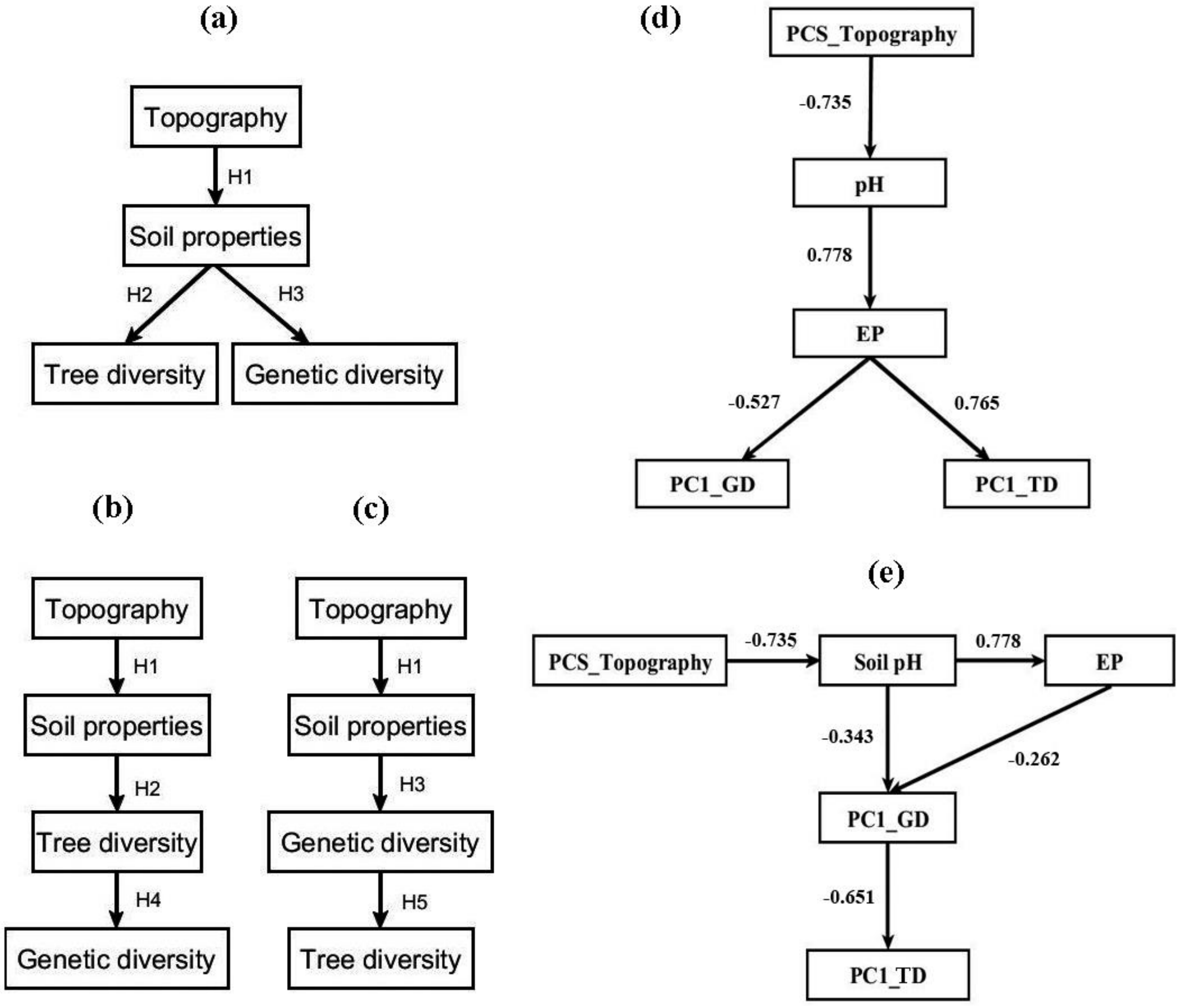

Figure S4 Conceptual models with hypothesized connection pathways from the structural equation modeling analysis, with two alternative SEM models. We hypothesize that topography primarily affects soil properties (e.g. soil nutrients or pH) (H1)[2] and that soil properties (particularly the availability of limiting nutrients) affect tree diversity and genetic diversity of *B. roxburghiana* in parallel (H2 and H3; Conceptual model a). Simultaneously, the increase in tree diversity within the community could also negatively affect the genetic diversity of *B. roxburghiana* by reducing the breadth of the average niche or through other mechanisms (H4) and therefore leads to a negative correlation between tree diversity and genetic diversity (Conceptual model b). Moreover, the increased genetic diversity of a dominant tree species can also negatively affect the tree species diversity within the community (H5, Conceptual model c); this conceptual model is the least supported within our

study system because the individuals of *B. roxburghiana* were only 1.24% of the total number of trees within our plots and therefore were likely to have little effect on the other trees within the community. Two possible models were constructed and are shown here (Models d and e), but these models did not fit well to the primary data (Model d: CFI= 0.901; GFI =0.792; RMSEA = 0.220 and Model e: CFI= 0.787; GFI =0.786; RMSEA = 0.353), indicating that the two models were not appropriate.

Appendix S1 List of trees with a DBH>1 cm recorded across the plots. The ranking of the trees within the appendix was obtained by the abundance of the trees within the plots we studied.

Code	Trees
1	*Pittosporopsis kerrii*
2	*Parashorea chinensis*
3	*Knema furfuracea*
4	*Garcinia cowa*
5	*Saprosma ternata*
6	*Baccaurea ramiflora*
7	*Mezzettiopsis creaghii*
8	*Nephelium chryseum*
9	*Cinnamomum bejolghota*
10	*Dichapetalum gelonioides*
11	*Mallotus garrettii*
12	*Beilschmiedia roxburghiana*
13	*Semecarpus reticulata*
14	*Phoebe lanceolata*
15	*Pseuduvaria indochinensis*
16	*Ficus langkokensis*
17	*Trigonostemon thyrsoideum*
18	*Diospyros hasseltii*
19	*Syzygium latilimbum*
20	*Leea compactiflora*
21	*Barringtonia pendula*
22	*Dysoxylum binectariferum*
23	*Chisocheton siamensis*
24	*Sumbaviopsis albicans*
25	*Ficus fistulosa*
26	*Xanthophyllum siamense*
27	*Garcinia lancilimba*
28	*Harpullia cupanioides*
29	*Microcos chungii*
30	*Magnolia henryi*
31	*Aidia yunnanensis*
32	*Macropanax dispermus*
33	*Antidesma montanum*
34	*Pometia tomentosa*
35	*Knema globularia*
36	*Drypetes hoaensis*

37	*Epiprinus siletianus*
38	*Sloanea tomentosa*
39	*Dendrocnide sinuata*
40	*Cleidion brevipetiolatum*
41	*Lasianthus verticillatus*
42	*Ardisia thyrsiflora*
43	*Mallotus tetracoccus*
44	*Polyalthia simiarum*
45	*Castanopsis indica*
46	*Eurya austroyunnanensis*
47	*Sarcosperma kachinense var. simondii*
48	*Symplocos cochinchinensis*
49	*Pterospermum menglunense*
50	*Castanopsis megaphylla*
51	*Macaranga indica*
52	*Walsura robusta*
53	*Urophyllum chinense*
54	*Lasiococca comberi*
55	*Aquilaria yunnanensis*
56	*Antidesma japonicum*
57	*Cryptocarya acutifolia*
58	*Litsea dilleniifolia*
59	*Drimycarpus racemosus*
60	*Diospyros nigrocortex*
61	*Walsura yunnanensis*
62	*Sterculia lanceolata*
63	*Pygeum macrocarpum*
64	*Canarium tonkinense*
65	*Beilschmiedia robusta*
66	*Symplocos sp1*
67	*Turpinia pomifera*
68	*Dysoxylum sp1*
69	*Amoora duodecimantha*
70	*Gomphandra tetrandra*
71	*Aglaia perviridis*
72	*Mitrephora thorelii*
73	*Amoora yunnanensis*
74	*Orophea hainanensis*
75	*Gironniera subaequalis*
76	*Ardisia solanacea*
77	*Miliusa sinensis*
78	*Artocarpus tonkinensis*
79	*Mayodendron igneum*

80	*Oreocnide frutescens*
81	*Memecylon cyanocarpum*
82	*Syzygium rockii*
83	*Alphonsea monogyna*
84	*Alphonsea hainanensis*
85	*Oreocnide sp2*
86	*Garcinia xanthochymus*
87	*Phlogacanthus curviflorus*
88	*Neonauclea sp1*
89	*Metadina trichotoma*
90	*Sarcosperma kachinense*
91	*Platea latifolia*
92	*Litsea baviensis*
93	*Elaeocarpus austroyunnanensis*
94	*Horsfieldia kingii*
95	*Glycosmis lucida*
96	*Garuga floribunda var. gamblei*
97	*Engelhardtia spicata*
98	*Elaeocarpus rugosus*
99	*Diospyros xishuangbannaensis*
100	*Tarennoidea wallichii*
101	*Myristica yunnanensis*
102	*Garcinia xishuangbannaensis*
103	*Ficus sp1*
104	*Canarium subulatum*
105	*Beilschmiedia sp1*
106	*Persea tenuipilis*
107	*Ostodes katharinae*
108	*Litchi chinensis*
109	*Ficus chrysocarpa*
110	*Drypetes sp1*
111	*Beilschmiedia purpurascens*
112	*Actinodaphne henryi*
113	*Memecylon polyanthum*
114	*Mallotus sp1*
115	*Lindera metcalfiana*
116	*Horsfieldia glabra*
117	*Castanopsis echidnocarpa*
118	*Artocarpus lakoocha*
119	*Ardisia quinquegona*
120	*Saurauia tristyla*
121	*Phoebe puwenensis*
122	*Litsea verticillata*

123	*Horsfieldia pandurifolia*
124	*Carallia brachiata*
125	*Alseodaphne petiolaris*
126	*Neonauclea griffithii*
127	*Mangifera sylvatica*
128	*Eriobotrya prinoides*
129	*Croton sp2*
130	*Aporusa yunnanensis*
131	*Ambroma augusta*
132	*Syzygium oblatum*
133	*Glochidion lanceolarium*
134	*Elaeocarpus varunua*
135	*Dysoxylum hongkongense*
136	*Lithocarpus vestitus*
137	*Lasianthus chrysoneurus*
138	*Dolichandrone stipulata*
139	*Colona thorelii*
140	*Ailanthus triphysa*
141	*Vitex quinata*
142	*Tabernaemontana corymbosa*
143	*Schefflera bodinieri*
144	*Pavetta hongkongensis*
145	*Ormosia sp1*
146	*Oreocnide rubescens*
147	*Myrsine seguinii*
148	*Mitrephora maingayi*
149	*Microtropis discolor*
150	*Dysoxylum densiflorum*
151	*Castanopsis hystrix*
152	*Mallotus barbatus*
153	*Litsea pierrei*
154	*Ficus hispida*
155	*Duperrea pavettifolia*
156	*Beilschmiedia fasciata*
157	*Trevesia palmata*
158	*Syzygium cathayense*
159	*Phoebe minutiflora*
160	*Garuga pinnata*
161	*Ficus auriculata*
162	*Celtis biondii*
163	*Actinodaphne obovata*
164	*Trema orientalis*
165	*Saurauia cerea*

166	*Ficus variolosa*
167	*Ficus subincisa*
168	*Ficus esquiroliana*
169	*Elaeocarpus glabripetalus*
170	*Croton sp3*
171	*Cinnamomum tenuipilis*
172	*Alphonsea sp1*
173	*Aglaia abbreviata*
174	*Pterospermum lanceifolium*
175	*Mallotus paniculatus*
176	*Dysoxylum lukii*
177	*Drypetes perreticulata*
178	*Chisocheton paniculatus*
179	*Bridelia tomentosa*
180	*Apodytes sp1*
181	*Xanthophyllum yunnanense*
182	*Sapium baccatum*
183	*Meliosma kirkii*
184	*Litsea panamanja*
185	*Litsea garrettii*
186	*Lithocarpus grandifolius*
187	*Linociera insignis*
188	*Lagerstroemia tomentosa*
189	*Horsfieldia tetratepala*
190	*Heteropanax fragrans*
191	*Flacourtia ramontchii*
192	*Ficus hirta*
193	*Elaeocarpus sphaerocarpus*
194	*Elaeocarpus apiculatus*
195	*Diospyros kaki*
196	*Chukrasia tabularis*
197	*Bridelia insulana*
198	*Bischofia javanica*
199	*Alchornea tiliifolia*
200	*Trichilia connaroides*
201	*Terminalia bellirica*
202	*Schima wallichii*
203	*Radermachera microcalyx*
204	*Olea rosea*
205	*Manglietia forrestii*
206	*Macropanax undulatus*
207	*Homalium ceylanicum var. laoticum*
208	*Ficus oligodon*

209	*Ficus glaberrima*
210	*Euodia austrosinensis*
211	*Dendrocnide urentissima*
212	*Dalbergia sp1*
213	*Croton argyratus*
214	*Castanopsis mekongensis*
215	*Canthium simile*
216	*Alstonia rostrata*
217	*Ailanthus fordii*
218	*Tapiscia yunnanensis*
219	*Syzygium yunnanense*
220	*Pavetta polyantha*
221	*Oxyceros sinensis*
222	*Mussaenda macrophylla*
223	*Mitrephora wangii*
224	*Litsea monopetala*
225	*Glycosmis pentaphylla*
226	*Ficus cyrtophylla*
227	*Cryptocarya sp1*
228	*Croton kongensis*
229	*Calophyllum polyanthum*
230	*Aquilaria sp1*
231	*Aphananthe cuspidata*
232	*Alstonia scholaris*
233	*Acer garrettii*
234	*Xanthophyllum sp1*
235	*Ulmus lanceifolia*
236	*Tetrameles nudiflora*
237	*Syzygium cumini*
238	*Sapindus rarak*
239	*Pseuderanthemum malaccense*
240	*Pouteria grandifolia*
241	*Phoebe sp1*
242	*Paramichelia baillonii*
243	*Meliaceae sp1*
244	*Mallotus philippinensis*
245	*Litsea sp2*
246	*Laurocerasus phaeosticta*
247	*Homalium sp1*
248	*Ficus sp3*
249	*Ficus benjamina*
250	*Euodia glabrifolia*
251	*Elaeocarpus sp1*

252	*Elaeocarpus prunifolioides*
253	*Debregeasia longifolia*
254	*Dalbergia sp2*
255	*Cylindrokelupha yunnanensis*
256	*Croton sp1*
257	*Cinnamomum chartophyllum*
258	*Aphananthe aspera*
259	*Anthocephalus chinensis*
260	*Adenanthera pavonina*
261	*Turpinia montana*
262	*Toona ciliata*
263	*Syzygium fruticosum*
264	*Stereospermum colais*
265	*Scleropyrum wallichianum*
266	*Schefflera fengii*
267	*Pygeum topengii*
268	*Pandanus furcatus*
269	*Oreocnide sp1*
270	*Mussaenda multinervis*
271	*Morus macroura*
272	*Morinda angustifolia*
273	*Melia toosendan*
274	*Medinilla septentrionalis*
275	*Manglietia sp1*
276	*Mangifera sp1*
277	*Macaranga henryi*
278	*Litsea sp1*
279	*Litsea euosma*
280	*Lithocarpus truncatus*
281	Lauraceae *sp1*
282	*Lasianthus rigidus*
283	*Gmelina arborea*
284	*Ficus sp2*
285	*Ficus semicordata*
286	*Ficus sagittata*
287	*Ficus altissima*
288	*Euodia sp1*
289	*Engelhardtia roxburghiana*
290	*Elaeocarpus sp3*
291	*Elaeocarpus sp2*
292	*Dysoxylum sp2*
293	*Diplospora mollissima*
294	*Dalbergia fusca*

295	*Cinnamomum tamala*
296	*Cinnamomum mollifolium*
297	*Cinnamomum glanduliferum*
298	*Celtis timorensis*
299	*Celtis philippensis*
300	*Capparis sabiaefolia*
301	*Callicarpa bodinieri*
302	*Broussonetia papyrifera*
303	*Brassaiopsis glomerulata*
304	*Aporusa villosa*
305	*Apodytes dimidiata*
306	*Amoora sp1*
307	*Albizia procera*
308	*Albizia chinensis*
309	*Aidia sp1*
310	*Aglaia sp1*
311	*Acrocarpus fraxinifolius*

Reference

1. Liu, S. L. *et al*. The relationship between landform, soil characteristics and plant community structure in the Danglingshan mountain region, Beijing. *Acta Phytoecol. Sin.* **27**, 496-502 (2003).

2. Sollins, P. Factors influencing species composition in tropical lowland rain forest: does soil matter? *Ecology* **79**, 23-30 (1998).
